# Use and Misuse of Material Transfer Agreements: Lessons in Proportionality from Research, Repositories, and Litigation

**DOI:** 10.1371/journal.pbio.1002060

**Published:** 2015-02-03

**Authors:** Tania Bubela, Jenilee Guebert, Amrita Mishra

**Affiliations:** School of Public Health, University of Alberta, Edmonton, Alberta, Canada

## Abstract

Material transfer agreements exist to facilitate the exchange of materials and associated data between researchers as well as to protect the interests of the researchers and their institutions. But this dual mandate can be a source of frustration for researchers, creating administrative burdens and slowing down collaborations. We argue here that in most cases in pre-competitive research, a simple agreement would suffice; the more complex agreements and mechanisms for their negotiation should be reserved for cases where the risks posed to the institution and the potential commercial value of the research reagents is high.

## Abstract 2

The material transfer agreements designed to facilitate the exchange of materials between researchers are unnecessarily burdensome and obstructive and in most cases could be replaced by simpler tools.

## Introduction

A material transfer agreement (MTA) is a type of legally enforceable contract employed by research institutions and companies to set the terms under which their materials and associated data may be obtained and used by others (Box 1) [1,2]. These agreements provide a mechanism to protect the interests of the owners of discoveries and inventions, while promoting data and material sharing in the research community. The latter is an admirable goal in an age where research is increasingly collaborative, multinational, and multidisciplinary. Yet MTAs have a bad reputation among researchers for being overly complex and, in practice, hindering the exchange of research reagents. This article examines what is wrong with current practices in negotiating and drafting MTAs; it suggests a way forward in which the complexity of the agreement should reflect the likely risks and benefits of sharing hard-earned reagents with other researchers.

**Box 1. Anatomy of an MTA**

MTAs may apply to anything from materials that are simply under the control of the originator but have no formal intellectual property rights attached to them to proprietary materials protected by patents and trade secrets [3,4]. They range in complexity from simple conditions of use to complex legal agreements [5] with many terms [3–5]. They may comprise standard form agreements or they may be specifically negotiated. Broadly speaking, MTAs delimit the rights and responsibilities of the providers and recipients of materials, and they describe the materials to be transferred and the payment or other benefit to be exchanged in return. As such, they conform to standard common law requirements for legally enforceable contracts: an intention to create a legal relationship, a “meeting of the minds” (offer and acceptance of terms), and an exchange of consideration (e.g., money, goods, and/or services). MTAs commonly place limits on the use of materials, their physical handling, and distribution to third parties. For example, the use of materials may be limited to noncommercial or preclinical research, or to specific fields of use, such as research on a specific disease. Distribution to third parties may be prohibited, or subject to permission from the provider. Other standard terms limit the liability of providing institutions with respect to the quality of the materials and any third party intellectual property rights that may have been infringed in their creation. They also outline dispute resolution mechanisms, legal jurisdiction, the duration of the relationship, and conditions for its termination, including the return or destruction of the materials. MTAs sometimes also contain problematic “reach-through” terms, however, that lay proprietary claims on subject matter developed *using* the material or that incorporates the material.

## The Problem with MTAs As Used by Research Institutions

Research staff and students usually lack both the knowledge and legal authority to enter into contracts, like MTAs, on behalf of their institutions. To address this lack of capacity, most institutions have offices with a dedicated staff to negotiate, draft, and execute MTAs [2,5]. This centralization of contract services in a technology transfer or research services office is problematic because it can lead not only to delays but also to conflicts between the interests of researchers and the interests of their institutions. Researchers commonly express frustration with institutional processes [6]. Surveys and interview-based studies of researchers have come to the conclusion that access to research reagents is hampered by negotiations over MTAs, whose complexity rarely reflects the value to the institution of the materials to be shared [7–9]. There is a cultural divide between research communities, which may simply want to share their data and materials, and institutional contracts or technology transfer staff. The latter are in the difficult position of trying to fulfill two mandates: first, to commercialize the research at their institution to achieve a financial return on investment and, second, to promote the desired community-level sharing of materials and data [10–12].

A further criticism of MTAs is that their terms may expand the rights of the institution well beyond those granted under formal intellectual property laws [13,14] in the following ways. First, MTAs may control the use of materials that would usually not be eligible for intellectual property protection or where such intellectual property has expired. Second, MTAs may set limits on use in countries where the inventors or owners have not sought or been granted patent protection. Third, and most problematic, MTAs may grant rights over patented inventions far beyond those contemplated by patent policy. A patent is a bargain between the inventor(s) and society: inventors gain the right to exclude others from exploiting an invention for a limited period (usually 20 years); in exchange, society gains knowledge by way of a detailed description of that invention. The terms of an MTA may, however, extend rights beyond the patented invention if they “reach through” the patent to lay claim (e.g., for royalties) on anything developed using the invention or that incorporates the invention. Examples include inventions that incorporate a patented nucleotide or amino acid sequence, or an invention developed using a common method or reagent. Such reach-through rights are discouraged by most policies and guidelines for best practice in licensing university-generated innovation [15,16]. These best practice policies and guidelines should equally apply to MTAs, because MTAs are a type of license (a permission to use) where the subject matter of the license is material and associated data [17].

## Lessons from Mouse Research

Public funding agencies invest considerable resources in the generation of research reagents, and the return on this investment is maximized when those reagents are shared. Sharing avoids duplication of effort in the development of reagents and enables replication and further innovation based on published methods. The United States’ National Institutes of Health (NIH) has been particularly proactive in developing and instituting policies for the sharing of materials [18–20]. These policies were, in part, a response to restrictions over access to two transgenic mouse technologies: OncoMouse, a mouse strain with a genetic predisposition to cancer, developed by researchers at Harvard and exclusively licensed by DuPont; and Cre-lox, a technology for generating conditional mouse mutants, developed by DuPont researchers [21,22]. In both cases, the NIH stepped in to negotiate access and distribution on less restrictive terms than the original MTAs proposed by DuPont [23–25].

NIH policy from 1999 directs that research reagents generated through the use of public funds be transferred amongst researchers either with “no formal agreement, a cover letter, the Simple Letter Agreement of the Uniform Biological Materials Transfer Agreement (UBMTA), or the UBMTA itself” (Box 2) [19,26]. Nevertheless, estimates suggest that only approximately 35% of mouse strains are made available to the research community [27]. This deficit is due in part to scientific competition: researchers want to retain priority in their field, and they fear being scooped by other researchers using their materials or data. Also, distribution of materials to research groups on request can be expensive in terms of the resources and time involved, especially for popular research reagents. Although research archives like biorepositories, biobanks, and databases can relieve the burden of distribution from research groups, there are still many direct and indirect costs associated with preparing the materials for deposit.

**Box 2. Simplified Models for Sharing Materials**

In March 1995, the NIH put in place a simplified system for sharing nonproprietary materials amongst research institutions [26]. To date, 532 institutions, mainly in the US, are signatories to this UBMTA Master Agreement, meaning that materials can be transferred amongst researchers at those institutions upon execution of a simple implementing letter that provides a record of the transfer. The UBMTA applies to the material, progeny, and unmodified derivatives. Materials may be used for teaching and academic research that is not in human subjects and is compliant with all laws and policies governing research. There are no restrictions on publication. The materials may be used by the recipient and those working under his or her direction; transfer to third parties is prohibited. Standard warranties and liabilities apply, including that the materials may be the subject of patent rights or applications. The materials are provided on a cost recovery basis only. While the UBMTA is not perfect—the legal language could be more accessible, and some terms apply not only to the original material but also to modifications that contain or incorporate it—the UBMTA supports a simplified system for transfer. Other institutions, such as the University of California, transfer materials based on a modified UBMTA, which defeats the purpose of a unified distribution system by requiring review and signoff by recipient institutions.

Nonacademic organisations that distribute research reagents also use mechanisms based on the UBMTA. For example, Addgene, a nonprofit plasmid repository, uses an implementing letter accompanied by an MTA for nonprofit or academic institutions [28]. While the MTA can be emailed or mailed, Addgene has implemented a novel, proprietary, and award-winning electronic MTA (eMTA) system, which handles the majority of agreements [29]. The system is designed to accept online signatures and to be simple to use for technology transfer officers at depositing and requesting institutions. In addition, unlike the University of California, Addgene does not allow "red lining" or editing of its MTA. The depositors all use the same MTA, and the recipients must E-sign as is or the material cannot be accessed. This saves time on legal wrangling over terms for materials with little value outside of the research context.

Other simplified MTA distribution systems also exist, such as those of The Jackson Laboratory (JAX) [30] and the Structural Genomics Consortium (SGC), a not-for-profit public—private partnership that provides 3-D structures of biologically significant human proteins and probes for drug development [31]. It distributes probes under an MTA with four clauses stating use for laboratory research (not in humans or non-laboratory animals) only in compliance with national laws, for teaching, and not-for-profit research purposes. The SGC retains rights to the materials, encourages publication in the scientific literature, and prohibits transfer to third parties.

JAX embodies the NIH material sharing policy [27,30]. As one of the world’s oldest biomedical archives for mouse-related reagents, JAX both generates its own resources and accepts mice from research groups after an assessment of their novelty and quality. Many lessons may be learned from the JAX model. Because of JAX’s reputation as a public archive, it can set terms and conditions on deposit that foster a culture of sharing in the community. These terms enable JAX to distribute mouse strains to academic and not-for-profit researchers under a simple notification that the mice are for research purposes and are not for sale or transfer to third parties without permission [32,33]. In addition, JAX enables depositors to decide the conditions under which to distribute mouse strains to industry, if at all [30]. By distributing mice to industry only when it has received notification of an executed MTA between the industry recipient and the depositor’s institution, JAX acts as a broker, thus maintaining the trust of the research community [6,33].

Another example from the mouse research community is the International Knockout Mouse Consortium (IKMC) and the associated International Mouse Phenotyping Consortium (IMPC), which were created and supported by several international funding agencies. The IKMC is generating mutants in all protein-coding mouse genes in a standard mouse strain, and the IMPC is phenotyping a substantial proportion of these using standardized protocols [34,35]. Both consortia rely on new and established archives to distribute this high throughput resource [35]. One of the IKMC repositories at the University of California at Davis distributes portions of the resource to both academia and industry using an MTA closely modeled on the UBMTA (Box 2) [36]. This is made possible by a special provision in its resource development contract from the NIH, known as *Authorization and Consent* [37]. This provision insulates the resource generators located in the US, as government contractors, from potential patent infringement litigation over background intellectual property over the myriad technologies used to create the resource [37]. However, this protection is not available in other countries. The European repositories that are part of the consortium, in particular, have found it difficult to distribute to industry because of fears of patent infringement claims over background intellectual property. In addition, differences in legal cultures and drafting styles make European MTAs more cumbersome than their US counterparts [6]. These factors impede distribution to the research community at large and hinder sharing of mouse lines amongst consortia members. Sharing among the consortia’s repositories is needed not only to accelerate phenotyping efforts, but also to alleviate issues with cross-border distribution, biosecurity for the resources, and sustainability of the repositories.

**Figure 1:**
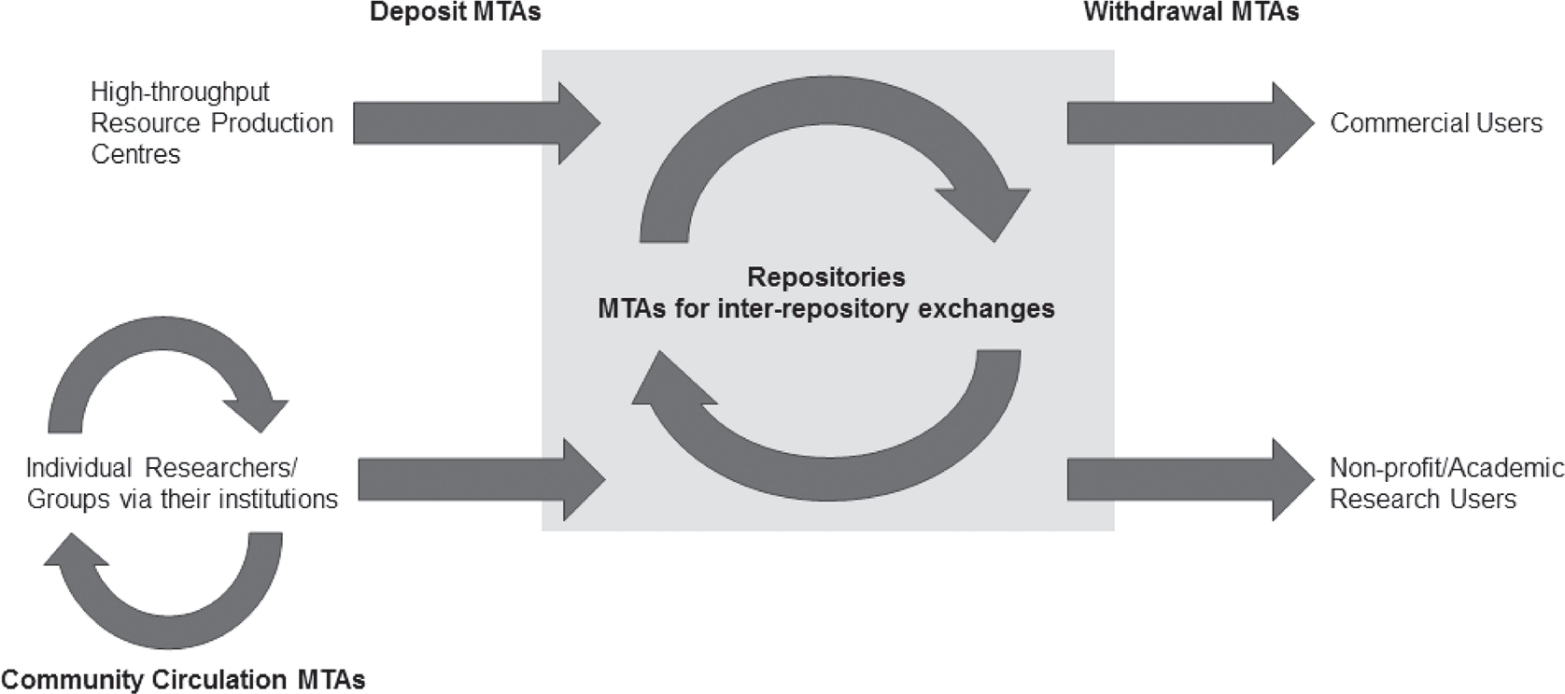
MTAs mediate exchanges amongst researchers for the deposit of materials into repositories, for the distribution of those materials to users, and for exchange of materials amongst repositories. The terms of deposit may set the terms for subsequent withdrawal. Variation in deposit terms requires considerable informatics infrastructure to manage and apply terms for later withdrawal. The system is therefore streamlined when deposit terms are constant for the bulk of resources in repositories. Note that repositories may also act as brokers between depositors and users, only distributing upon provision of evidence of an executed MTA.

The lesson for the IKMC and the IMPC is that simple and uniform conditions for depositing mutant mice in archives, such as those employed by JAX, have a knock-on effect enabling equally simple conditions for their distribution (Fig. 1), thereby reducing the transactional costs and complexities associated with the sharing of research reagents. Indeed, leading research universities in North America reduce the administrative burden even further by not using MTAs when exchanging nonhazardous or nonhuman biological materials. In other circumstances, research institutions may use standard agreements, such as the UBMTA, or Simple Letter Agreement, to enhance administrative efficiency (Box 2). There are some benefits to the latter approach in accurate record keeping of the use of research reagents (a metric for research impact akin to the citation of a publication) and identification of potential collaborators for the developers of the research reagents.

Additional complexities arise, however, when the transfer of materials involves human samples [38,39]. Informed consent given by research participants determines the use of their samples; for example, limiting research to a specific disease. Thus, if each sample in a biobank is collected using a different consent form, the samples may be deposited on different terms. Those terms must then attach to the sample and, in turn, dictate the distribution terms. This adds a layer of complexity to the transactions managed by biobanks, requires significant informatics resources, and may impede the ability of biobanks to accept legacy materials and data from the research community. Additional constraints arise for associated data that may link to patient records or other identifiable information. In this case, MTAs must comply with national or regional privacy laws in setting conditions for storage and use of samples and associated data. In other words, managing legal interoperability may be as problematic as managing data or metadata interoperability. Simplified practices and standardised legal terms, including consent, for input and distribution reduce these problems considerably.

## Lessons from Litigation

Legal action is costly in financial terms as well as in human energy and emotion [40], so much so that instances of litigation can serve as a metric for assessing the value of the issue to the parties. For example, litigation is a metric for the value of a patented invention [41] or the value of a contract, with real estate, employment, and construction contracts commonly litigated. This begs the question, do MTAs have any value as measured by the number of cases of litigation? When we searched for variations of the phrase “material transfer agreement” in the Westlaw US Premier Service (all Federal, Supreme Court, District Court, and State cases); Westlaw’s United Kingdom (UK) Law database; and the EUR-Lex database for European cases, we found only 23 cases, and one additional case in the Lex-Machina database, which covers intellectual property litigation that has been filed in a state or federal non-appellate level court (see S1 Text for a summary of the 24 sets of litigation) [42]. Even if our search missed some cases, clearly the proportion of material or data transfers that ever results in litigation is extremely small. Two contrary conclusions may be drawn from this: either MTAs are so well drafted and monitored that litigation is avoided—in other words, MTAs effectively discourage litigation—or institutional contracts staff or offices lack the resources to monitor and enforce the terms of their MTAs and, in general, the value of their subject matter is low. In our opinion, this latter explanation is most likely.

The 23 cases of litigation we identified fell into two categories: first, direct litigation, which claims a breach of the terms of the MTA, including confidentiality provisions (four cases) or a related action, such as unjust enrichment, where one party unjustly benefits at the expense of the other, giving rise to an obligation to make restitution even in the absence of wrongdoing (one case); and, second, indirect litigation, in which the MTA is used as evidence in a larger lawsuit (18 cases). Some of these cases are related to technical points of law; for example, the very existence of an MTA between two parties may be used as evidence to prove that a party has a legal presence in a particular jurisdiction (e.g., a US State) so that a particular court can hear the dispute. In a related use, a specific term in an MTA about how disputes are to be resolved may dictate the appropriate jurisdiction (e.g., a specific district court) in a broader dispute. The presence or absence of an MTA may be evidence of standard, university, company, or industry practice; for example, the MTA may be evidence of whether research was done as part of a collaboration or as part of sponsored research, which distinction has implications for identifying inventor(s) on a patent, and the owner of those patent rights. In that vein, the terms of an MTA may provide evidence of ownership of materials, inventorship of a patent, or contain an obligation to assign (transfer) patent rights. Ownership of patents and/or the existence of a license to use a patented invention are both important considerations in patent infringement litigation and may serve as a defence.

Only six cases concerned corporate parties with no involvement of a research institution or affiliated researcher. In all the other cases, litigation occurred between research institutions, institutions and their employees, and institutions and corporations. The cases were dominated by biomedical applications, with only six cases in other fields, including biofuels and agriculture. Some cases were particularly vitriolic and generated negative comments against some parties. Without doubt, these legal disputes had the potential to damage research relationships and the reputations of all actors involved.

Two cases stood out that may cause concern for the research community. One researcher at the University of Pittsburgh was criminally prosecuted for mail fraud for ordering microbial materials from the American Type Culture Collection using his institutional approved account on behalf of a fellow researcher at an unapproved institution, in violation of an MTA that prohibited transfer of materials to third parties [43]. The action was dismissed because of lack of evidence of misrepresentation, and the observation that the indictment did not allege that either of the researchers “even knew about the transfer restriction.” In the second case, the State University of New York attempted to dismiss an employee because he founded a company to sell and ship mice owned by Upstate Medical University, charged the shipping costs to a State University grant contract, and failed to comply with university MTAs [44]. The judge found that since he did not intend to profit personally, believed his company would benefit the University and provide financial support for his research, and the university administrators were aware of his actions, he should be suspended rather than dismissed [44]. Again, given the vast quantity of transactions over research tools in the US, these two cases do not imply significant risks for researchers.

Box 3 summarizes broad lessons from litigation on when complex and carefully negotiated MTAs are warranted. These circumstances indicate where the focus and capacity development for institutional contracts staff and offices should lie. These may otherwise be overwhelmed by MTAs in contexts where simpler mechanisms for exchange exist and are more appropriate.

**Box 3. When Might Complex MTAs Be Warranted?**

Lessons may be drawn from litigation on when more complex MTAs might be warranted. In keeping other uses of MTAs as simple and standardized as possible, the human and financial capacity of institutional contracts offices may be directed where they are most needed. MTAs should be used in transfers of materials to industry, especially when an industry partner is evaluating material during negotiations of broader licensing agreements [45–49]. MTAs should also be used for materials that will be used in clinical or commercial development [45–47]. In the latter context, given that development trajectories are often uncertain, MTAs should include clear definitions and terms that trigger an obligation to renegotiate the MTA if the nature of the research collaboration changes; for example, from preclinical to clinical or from noncommercial to commercial research [50–53]. In ongoing litigation over chimeric antigen receptors (CARs) between the University of Pennsylvania (Penn) and St. Jude Children’s Research Hospital, problems arose when the definition of a modified derivative in an MTA did not change when the use of a CAR by researchers at Penn changed from preclinical to clinical research [50–53]. In a case such as this, especially when pharmaceutical and biotechnology companies become involved in the research, reusable, standard, “boilerplate” terms are not acceptable. However, avoidance of problems and navigation of complex research relationships requires expertise in institutional contracts offices for negotiating, drafting, and monitoring the terms of agreements. Such expertise is often lacking.

In keeping with general observations on reach-through rights, research institutions should beware of industry agreements that contain expansive proprietary and licensing rights in favour of the industry partner [54].

Since MTAs may play an important role in determining ownership on patent applications and the assignment of proprietary rights, MTAs should fairly and clearly recognize the respective contributions of the parties [55,56]. Research institutions need to assist employees in navigating their relationships with industry, or they may lose valuable intellectual property rights [57–59]. When materials have been widely distributed to the research community in the absence of an MTA, it is usually too late for an institution or researcher to claim proprietary rights and benefits from the user community [60]. In these circumstances, launching litigation is costly for all parties.

## The Shape of MTAs to Come

The lessons learned from materials and data sharing in the mouse research community and from the rare instances where research relationships failed and litigation ensued suggest some core principles upon which to build the MTAs of the future.

1. Keep it simple: The core message from the mouse community is that both the deposit and distribution of MTAs should be kept as simple as possible, so that institutions can realistically monitor and enforce the terms. MTAs should also be commensurate with a realistic assessment of the risks and benefits to the institutions, both in terms of legal liabilities and potential revenue generation. In the case of research tools, the benefits are rarely monetary; the main benefits arise from the use of research reagents to advance the scientific field, giving due acknowledgement of their source.
2. Management of risks should be proportionate to the type and likelihood of benefits: Institutional contracts staff should evaluate the presence or likelihood of the following categories of risks relative to the likely benefits to accrue to the institution and/or its researchers and use MTAs or simpler agreements accordingly. First, risks relate to safety in the use of the materials; for example, ensuring that the research-grade materials are not used in research on human subjects, or that materials are used in compliance with ethical standards. A second class of risk is legal: inappropriate use of reagents may result in legal action by victims against institutions; third parties may claim that the material infringes their intellectual property rights, or that the materials supplied are not of the quality claimed by the distributor. A third class of risk is to reputation: MTAs may require appropriate acknowledgment of the originator or the distributing archive in any ensuing publications or other research outputs, so promoting the reputation of the source and providing a metric for the value of the material. The final category of risk is that third parties misappropriate the credit or financial reward that should accrue to the originator of the material: MTAs may limit distribution of reagents or materials to third parties, ensuring that users return to the originator to access materials under its terms, or may limit commercial use of the materials. These standard concerns are covered under the UBMTA and similarly simple agreements.
3. Avoid reach-through claims: Problems arise when institutions attempt to overreach their interests in an invention using reach-through claims [6]. These terms are problematic not only in terms of intellectual property policy but also from a negotiation perspective, and they will likely delay the execution of a final agreement. These terms are almost impossible to enforce, and therefore are of limited benefit. A policy against reach-through should apply for all MTAs—research institutions should be very wary of such terms that commonly attach to research materials shared by industry. Equally, institutions should not be tempted to insert reach-through provisions into MTAs, even if only attempting to promote access to research tools. While it may be good practice, especially in all forms of licensing agreements with industry, to retain rights in materials for further research, it is problematic to insist on an expanded set of rights to research using derivative materials. Indeed, such a provision in the original MTA for the distribution of European resources within IKMC was perceived to be highly problematic for international partners [6]. Here, systemic problems arise when deposit MTAs, or MTAs that share a resource with members of a consortium, contain reach-through provisions that need to be enforced by the receiving institution on behalf of the originating institution. In this scenario, the receiving institution derives no direct benefits, but is contractually obliged to enforce rights on behalf of another institution.

In conclusion, since MTAs are central mediators in the exchange of data and materials generated using public funds, they should embody policies and guidelines that encourage sharing and foster related community norms. Currently, complex MTAs and protracted negotiations create transactional bottlenecks that frustrate sharing, and they are unlikely ever to be monitored and enforced. Standard and simple agreements, by contrast, decrease the administrative burden for researchers, their institutions, and repositories. Institutions should adopt these simple agreements in cases where the risk is low and the benefits are noncommercial. They can then focus their energies on those cases where more complex agreements are warranted, especially in relations with industry and in contexts closer to commercial development and/or clinical application.

## Supporting Information

S1 Text MTA case summaries. (PDF)

## Abbreviations

MTA: Material transfer agreement
NIH: National Institutes of Health
UBMTA: Uniform biological materials transfer agreement
eMTA: electronic material transfer agreement
JAX: Jackson Laboratory
SGC: Structural Genomics Consortium
IKMC: International Knockout Mouse Consortium
IMPC: International Mouse Phenotyping Consortium
CAR: Chimeric antigen receptor
