# Supplementary material for: Use and Misuse of Material Transfer Agreements: Lessons in Proportionality from Research, Repositories, and Litigation
Source: PLoS Biol. 2015 Feb 3;13(2):e1002060. doi: 10.1371/journal.pbio.1002060 (PMC4315468; doi:10.1371/journal.pbio.1002060)
Supplement: S1 Text — (PDF) [file pbio.1002060.s001.pdf]

Supplemental Results: Use and Misuse of Material Transfer Agreements: Lessons in Proportionality from Research, Repositories and Litigation

Tania Bubela, Jenilee Guebert, Amrita Mishra

All authors are at the School of Public Health, University of Alberta, Edmonton, Alberta, Canada
Correspondence: tbubela@ualberta.ca

JUDICIAL CASE SUMMARIES

Between research institutions and research institutions and corporations

1. ***Stanford University v. Roche Diagnostics***

- Functional Genetics Inc. v. The Board of Trustees of the Leland Stanford Junior University, Civil Action No. 3:09-cv-04703-BZ (N.D. Cal. 2009).
- Board of Trustees of the Leland Stanford Junior University, Thomas Merigan, and Mark Holodniy v. Roche Molecular Systems Inc., Roche Diagnostics Corporation, and Roche Diagnostics Operations Inc. 583 F.3d 832 (Fed. Cir. 2009).

The Supreme Court of the United States (SCOTUS) decision in *Stanford v. Roche* stands for the proposition that the Bayh-Dole Act does not automatically confer ownership of federally-funded inventions to contractors, including research institutions that receive federal funds.[1] The Act does not authorize such contractors to unilaterally take title to inventions; instead patent rights always vest first in the inventor. This decision, therefore, has important implications for *who* can assign (transfer) ownership in federally funded inventions and how such assignment may operate through contracts, including Material Transfer Agreements (MTAs). Indeed the case engaged multiple MTAs and a Visitor Confidentiality Agreement (VCA).

Stanford, as the assignee of three patents for assessing the levels of human immunodeficiency virus (HIV) in blood samples, sued Roche Molecular Systems, the manufacturer of HIV detection kits, for patent infringement. Roche defended the suit by claiming that, as a co-owner of the patents, it could not be sued by Stanford. The facts revolved around the relationship between Stanford; a Stanford Research Fellow, Mark Holodniy; his supervisor, Thomas Merigan; a biotechnology company, Cetus that had developed the Polymerase Chain Reaction (PCR)[2]; and Roche, which later purchased Cetus' PCR assets and business. Holodniy had signed an employment agreement with Stanford that contained a promise to assign any inventions to Stanford. As a visiting researcher at Cetus to learn PCR and work on developing a PCR-based assay for HIV, he also signed a VCA that assigned the rights to Cetus. The Court of Appeals for

[1] Board of Trustees of the Leland Stanford Junior University v. Roche Molecular Systems, Inc., et al. 131 S. Ct. 2188 (S. Ct. 2011).
[2] Fore J, Joe; Wiechers, R., Ilse; Cook-Deegan, Robert (2006) The effects of business practices, licensing, and intellectual property on development and dissemination of the polymerase chain reaction: case study. Journal of Biomedical Discovery and Collaboration 1: 1-17.

the Federal Circuit (CAFC) found that the VCA assignment, expressed in the present tense ("does hereby assign"), trumped the promise of a future assignment to Stanford ("agrees to assign").[3] This issue was not on appeal to SCOTUS although the dissent expressed concerns with the CAFC's interpretation of the contractual language. Justice Breyer stated "[w]hile the cognoscenti may be able to meet [the CAFC's drafting rule] in future contracts simply by copying the precise words blessed by the Federal Circuit, the rule nonetheless remains a technical drafting trap for the unwary."[4]

At the trial level, MTAs between Merigan and Cetus also bolstered Cetus' claims to an ownership interest in the inventions.[5] Merigan was both a Professor of Medicine at Stanford and a member of Cetus' Scientific Advisory Board, with formal consulting agreements in 1980, 1984, and 1991. These agreements recognized Merigan's obligations to Stanford, however, Merigan also entered into a number of MTAs that enabled him "to use Cetus' proprietary materials and information in exchange for a non-exclusive, royalty-free license to Cetus for any intellectual property developed as a result of the MTA".[6] Merigan and Cetus shared patient samples, PCR protocols, and the results of PCR testing. The 1988 MTA provided that Stanford would inform CETUS of research results with the shared materials; identify Cetus' role in any commercially useful inventions; provide Cetus with copies of disclosures of inventions; and give Cetus the first option to an exclusive license with royalty terms to be negotiated in good faith, or alternatively, a non-exclusive license. The trial judge interpreted this last point to leave the choice between the latter alternatives to Cetus. In addition, the MTA applied to all researchers at Stanford, including later research efforts by Holodniy, because Stanford was an institutional signatory to the agreement. Thus Holodniy's "receipt and use of the materials in connection with Stanford's development of the PCR assay method was sufficient to trigger the licensing provision and grant Cetus a nonexclusive license in the patented inventions".[7] The MTA was, however, not considered in further litigation of the MTA because its wording did not allow Cetus to assign its nonexclusive license in the patented inventions to Roche. The MTA was therefore immaterial to later litigation.

The Lessons learned here in the context of MTAs are manifold. Employment contracts need to be carefully worded to ensure that inventions created in the normal course of employment are

---

[3] Board of Trustees of the Leland Stanford Junior University, Thomas Merigan, and Mark Holodniy v. Roche Molecular Systems Inc., Roche Diagnostics Corporation, and Roche Diagnostics Operations Inc. 583 F.3d 832 (Fed. Cir. 2009).
[4] Board of Trustees of the Leland Stanford Junior University v. Roche Molecular Systems, Inc., et al. 131 S. Ct. 2188 (S. Ct. 2011) – dissent.
[5] Functional Genetics Inc. v. The Board of Trustees of the Leland Stanford Junior University, Civil Action No. 3:09-cv-04703-BZ (N.D. Cal. 2009).
[6] Functional Genetics Inc. v. The Board of Trustees of the Leland Stanford Junior University, Civil Action No. 3:09-cv-04703-BZ (N.D. Cal. 2009).
[7] Functional Genetics Inc. v. The Board of Trustees of the Leland Stanford Junior University, Civil Action No. 3:09-cv-04703-BZ (N.D. Cal. 2009).

assigned to the research institutions. Without such assignment, other agreements, such as MTAs and confidentiality agreements, entered into by researchers with third parties, may trump the ownership interests of their employers. Research institutions need to monitor the wording in such agreements and support faculty in navigating the complex paperwork involved in research relationships with industry. Otherwise, even a Research Fellow may be able to sign away not only his own interests as a co-inventor, but also the interests of his institution in his portion of the invention.[8]

2. ***University of Pennsylvania v. St. Jude Children's Research Hospital***

- Trustees of the University of Pennsylvania v. St. Jude Children's Research Hospital, No. 12–4122, 2013 WL 5996864 (E.D. Pa. Apr. 12, 2013).
- Trustees of the University of Pennsylvania v. St. Jude Children's Research Hospital, 940 F.2d 233 (E.D. Pa. 2013).
- Trustees of the University of Pennsylvania v. St. Jude Children's Research Hospital, 940 F.2d 298 (E.D. Pa. 2013).
- Trustees of the University of Pennsylvania v. St. Jude Children's Research Hospital, 940 F.2d 518 (E.D. Pa. 2013).

This dispute between the University of Pennsylvania (Penn) and a research hospital (St. Jude's) is in its early stages in the United States District Court. To date, all actions have been consolidated and transferred to the Eastern District of Pennsylvania (November 2013). St. Jude filed a breach of contract action against Penn in July 2012 over two MTAs and eight days later, Penn filed a breach of contract action. In March 2013, St. Jude's patent over chimeric receptors was issued and three days later Penn filed a separate action seeking a declaration that it was not infringing on that patent and that the patent was invalid. The case is currently in the discovery stage and is scheduled for a jury trial. The tort case against St. Jude for interfering in contractual relationships between Penn and pharmaceutical company, Novartis, have been dismissed (April 2013), leaving claims of patent infringement and a contractual dispute over the interpretation of the terms of two MTAs. This summary focuses on the latter.

The MTAs arose out of research at St. Jude by Dario Campana and Chihaya Imai. In the early 2000s, Campana developed an anti-CD19 chimeric antigen receptor (CAR), which he inserted via a retroviral vector into T-cells to target and destroy cancer cells in chronic leukemia and non-Hodgkin's lymphoma. He presented his findings at an American Society of Hematology conference in 2003, after which Carl June at Penn wrote to him to suggest a collaboration. June suggested that a lentiviral vector might be safer and more efficacious vehicle to transduce T-cells. To facilitate the exchange, the parties, Penn and St. Jude, entered into an MTA, which defined the "Material" to be transferred from St. Jude to Penn as "the anti-CD19-BB-chimeric T-cell receptor construct, including any progeny, portions, *unmodified* derivatives and any accompanying know-how or data" [emphasis added]. The MTA provided that "the Material will

[8] Marshall E (2011) Signature on Visitor's Form Fuels Stanford v. Roche Court Battle. Science 332: 163.

only be used to create a lentiviral chimeric T-cell receptor construct to be used in pre-clinical studies", and "may not be used in humans" or "for any commercial purpose". Commercialization required prior written approval from St. Jude, this included notification of any patent applications that contained or incorporated the Material and associated methods. St. Jude provided the construct and after further correspondence, the gene sequence and the method to detect its expression.

June was one of the first researchers to work with lentiviral vectors for cancer immunotherapy. He and his team modified the sequence of the anti-CD19-BB-z for recombination with his lentiviral vector, resulting in a 5 nucleotide sequence difference at the ends and one PCR-related copy error within the sequence. Penn therefore described the June Construct as a "modified derivative" of the Campana Construct, while St. Jude concluded that "even with the base pair difference, the June Construct contains the largest possible nucleotide 'portion'". In 2007, St. Jude became aware that June may have sent the CAR to another researcher and that June was planning to conduct clinical trials. Penn confirmed the plans for the trial and St. Jude requested a new MTA for clinical use, which was executed in October 2007 but contained the same definition of "Material" as the 2003 MTA.

In April 2009, Campana and June co-authored an article in *Molecular Therapy* which acknowledged the Campana Construct generated at St. Jude.[9] However, when June published his clinical trial results, neither article acknowledged St. Jude as a source of material.[10] After a letter querying the lack of acknowledgement, June began to require third parties to seek permission from St. Jude before sending them his Construct "solely in order to avoid a legal dispute and out of an abundance of caution."[11] In November 2011, Penn informed St. Jude that it wished to terminate the MTA. Penn then began to negotiate with Novartis to develop June's cancer immunotherapy program, including 20 million USD towards a Center for Advanced Cellular Therapies. Novartis purchased a manufacturing plant to develop the first candidate CAR therapy for CTL019 cells, lentiviral vectors and CARs. St. Jude received a patent for a chimeric antigen receptor technology for cancer immunotherapies in July 2012. Three days later, Penn filed its action for non-infringement of the patent and St. Jude counterclaimed against Penn for willful infringement of its patent. At the same time, St. Jude has also been pursuing commercialization opportunities. Juno, a biotechnology company launched with 120 million USD by three prominent west-coast cancer research centers has licensed St. Jude's patent and has joined the

[9] Milone MC, Fish JD, Carpenito C, Carroll RG, Binder GK, et al. (2009) Chimeric Receptors Containing CD137 Signal Transduction Domains Mediate Enhanced Survival of T Cells and Increased Antileukemic Efficacy In Vivo. Mol Ther 17: 1453-1464.
[10] Porter DL, Levine BL, Kalos M, Bagg A, June CH (2011) Chimeric Antigen Receptor–Modified T Cells in Chronic Lymphoid Leukemia. New England Journal of Medicine 365: 725-733, 5. Amarnath S, Mangus CW, Wang JCM, Wei F, He A, et al. (2011) The PDL1-PD1 Axis Converts Human TH1 Cells into Regulatory T Cells. Science Translational Medicine 3: 111ra120.
[11] Trustees of the University of Pennsylvania v. St. Jude Children's Research Hospital, No. 12–4122, 2013 WL 5996864 (E.D. Pa. Apr. 12, 2013.

legal dispute against Penn (http://www.genengnews.com/gen-news-highlights/car-wars-startup-joins-st-jude-in-gene-therapy-legal-wrangle-vs-upenn/81249324/).

The patent dispute hinges on the interpretation of the two MTAs. St. Jude alleges that the June Construct *contains and was made with "Material"*. St. Jude claims that the Construct contains an almost *exact* copy, not a physical portion of the Campana Construct. Since the June Construct contains a gene sequence almost identical to the Campana Construct, the June Construct "contains" a portion of the anti-CD19-BB and is therefore subject to the commercialization and acknowledgement restriction of the MTAs. Penn, on the other hand, argued that "progeny, portions, unmodified derivatives and any accompanying know-how or data" did not cover the June Construct because it comprises a *modified* derivative. Other portions of the MTA support Penn's reading that the June Construct is not Material under the MTA and therefore is not subject to constraints on commercialization. They argue that use of the word "containing" rather than "incorporating" means that the MTA refers to *physical* portions of the Material only. Instead, the June Construct contains a modified derivative.

The judge found that whether the copy of the Campana Construct sequence in the June Construct constitutes a "portion" under the MTA is a matter of contractual interpretation. Since the plain language of the MTA is ambiguous and both interpretations are reasonable, and the conduct of both institutions, including ensuing correspondence, post MTA sheds no light on the interpretation, the interpretation cannot be determined by a judge alone in a preliminary motion and must go to full discovery and a jury trial.

Lessons learned: As the purpose of Penn's research with the Campana Construct changed from basic to clinical research with an industry partner and commercial intent, the wording and sophistication of the MTA should have reflected the changing relationship. St. Jude knew the exact use for the Campana Construct and tighter drafting and definitions, rather than boilerplate language, could have clarified that the lentiviral Construct contained a modified derivative of the Campana Construct in a manner that triggered St. Jude's proprietary interests. It could have clarified that it sufficed for the June Construct to contain portions of the same genetic sequence and not simply a *physical* portion of the Campana Construct. With a well-characterized research collaboration and specified intended use, there is no excuse to execute an MTA that is "facially ambiguous".

3. ***Massachusetts Eye and Ear Infirmary (MEEI) v QLT Phototherapeutics***

- Massachusetts Eye and Ear Infirmary v. QLT Phototherapeutics Inc. v. Massachusetts Eye and Ear Infirmary, Evangelos S. Gragoudas, M.D., Joan W. Miller, M.D., 412 F.3d 215 (1st Cir. 2005).
- Massachusetts Eye and Ear Infirmary v. QLT, 495 F. Supp 188 (D. Mass. 2007) [Trial decision, 2007].
- Massachusetts Eye and Ear Infirmary v. QLT Phototherapeutics Inc. v. Massachusetts Eye and Ear Infirmary, Evangelos S. Gragoudas, M.D., Joan W. Miller, M.D., 552 F.3d 47 (1st Cir. 2009) [CAFC En Banc]

In decade long legal dispute, confidentiality provisions within an MTA underpinned the MEEI's claim in unjust enrichment action against a biotechnology company spun out of the University of British Columbia. QLT held patents over a photosensitizer compound, benzoporphyrin derivative monoacid (BPD), derived from cow parsley. When a therapeutic property for destroying unwanted blood cells when exposed to targeted laser light was first discovered, "it was, for a time, a drug in search of a disease."[12] Through collaborators at Massachusetts General Hospital and later at MEEI, its potential for therapeutic use in the eye was discovered.

Dr. Joan Miller a fellow at MEEI obtained access to BPD through MTAs with QLT and showed primates that BPD could destroy abnormal choroidal blood vessels in the wet form of age-related macular degeneration without damaging healthy retinal vessels. "BPD had finally found its disease."[13] A series of MTAs and a confidentiality agreement provided Miller with BPD in preclinical research that formed the basis of a submission to the Food and Drug Administration (FDA). Dr. Miller also developed the clinical trial protocols and served as principal investigator until the FDA approved the BPD-based product to treat AMD, marketed as Visudyne.

While Visudyne was still in clinical trials, QLT approached CIBA Vision about a partnership for manufacturing and distributing the drug. QLT provided CIBA with confidential information from Dr. Miller, in clear breach of the material transfer and confidentiality agreements. It was clear that the partnership with CIBA was founded on this confidential information and further research results provided by Dr. Miller based on ongoing promises that QLT would negotiate a license on the ophthalmic use in good faith with MEEI. In a further series of maneuvers with respect to both the confidential information and patent filings, QLT reduced the value of MEEI's bargaining power and then offered a minimal royalty rate (0.5%) and upfront payment (a $200,000 research grant). The market for the drug was over 2.2 billion USD in net revenues. QLT's actions were sufficient for a jury, supported by two appellate decisions, to find that QLT was unjustly enriched at MEEI's expense and that its actions were "unfair and unscrupulous".

[12] Massachusetts Eye and Ear Infirmary v. QLT Phototherapeutics Inc. v. Massachusetts Eye and Ear Infirmary, Evangelos S. Gragoudas, M.D., Joan W. Miller, M.D., 552 F.3d 47 (1st Cir. 2009).
[13] Massachusetts Eye and Ear Infirmary v. QLT Phototherapeutics Inc. v. Massachusetts Eye and Ear Infirmary, Evangelos S. Gragoudas, M.D., Joan W. Miller, M.D., 552 F.3d 47 (1st Cir. 2009).

The jury awarded a past and future 3.01% royalty rate, equivalent to one eighth of the profits on the drug. As summed up by the District Court Judge, "The story of Visudyne is one of serendipity that would not have been possible without the contributions of every one of the researchers who entered the picture…The resulting treatment, Visudyne, should have been a cause for the celebration of the extraordinary contributions that each of these scientists made. Instead, the parties have squabbled over inventorship, minimizing and even dismissing outright contributions made by others…This dispute does not benefit the advancement of science, which builds on the contributions of all. Unless the parties can repair their relationships in the name of science, the losers may well be those who should have been the biggest winners – those who suffer from eye diseases."[14]

Lessons Learned: Again contractual provisions need to keep pace with the changing nature of relationships. The ducks need to be in a line when institutional partners are in a strong bargaining position. This cannot be done after the institutional partner's bargaining chips have been diminished.

4. ***Dana-Farber Cancer Institute, Inc. v. Gatekeeper Pharmaceuticals, Inc.***

- Dana–Farber Cancer Institute Inc. v. Gatekeeper Pharmaceuticals Inc. v. Dana–Farber Cancer Institute Inc. and Novartis Pharma A.G., Novartis International Pharmaceutical, Ltd., and Novartis Institutes for Biomedical Research, Inc., No. 10–11613, 2012 WL 4960172 (D. Mass. Oct. 12, 2012).

In 2012, Dana-Farber Cancer Institute sought a declaratory judgment in District Court against Gatekeeper Pharmaceuticals to establish the rights and responsibilities of each over a potential new therapeutic kinase inhibitor for drug-resistant small-cell lung cancers, WZ-4-002.[15] Researchers at Dana-Farber, a not-for-profit cancer research corporation discovered WZ-4-002, and Gatekeeper was founded by those researchers to rapidly advance the compound to clinical application. The issue was whether Gatekeeper Pharmaceuticals was entitled to license WZ-4-002 because of a pre-existing Collaborative Research Agreement (CRA) between Novartis and Dana-Farber. The CRA was very broadly worded to give Novartis priority in licensing the patents of Dana Farber principal investigators who received grant funding from Novartis. Novartis provided Dana Farber with between 8 and 10 million USD of research funding annually. Dana Farber initially decided that Novartis did not have priority to the compound, which enabled Gatekeeper to raise the requisite funds to license and develop it. However, Novartis, challenged this decision and Dana-Farber then gave Novartis priority, meaning that Gatekeeper lost its financial backers. Novartis then decided not to pursue the compound. The judicial motion was to determine whether Novartis in fact had priority or whether it interfered in

---

[14] Massachusetts Eye and Ear Infirmary v. QLT, 495 F. Supp 188 (D. Mass. 2007).

[15] Dana–Farber Cancer Institute Inc. v. Gatekeeper Pharmaceuticals Inc. v. Dana–Farber Cancer Institute Inc. and Novartis Pharma A.G., Novartis International Pharmaceutical, Ltd., and Novartis Institutes for Biomedical Research, Inc., No. 10–11613, 2012 WL 4960172 (D. Mass. Oct. 12, 2012).

Gatekeeper's contractual relationships. The Judge sided with Gatekeeper, stating: "nearly all scientific work is inspired by or based on information or ideas from other scientists. It cannot be that Novartis is entitled to a license to every invention ever made which is inspired by a discovery made during a Novartis-funded research project."

One of the arguments used unsuccessfully by Novartis was based on an MTA entered into by one of the Dana-Farber researchers, Michael Eck, who discovered WZ-4-002. The MTA was for a Novartis compound to study the structure of the kinases in question. The MTA prohibited the transfer to third parties, including other non-funded researchers at Dana-Farber, and the use for purposes other than the specified structural studies and to facilitate the optimization of kinase inhibitors. It the materials were used for other studies or transfer occurred, Novartis insisted on ownership of all patentable inventions and know-how over the materials "which are discovered or improved during the course of such unauthorized studies".[16] Eck shared information about the Novartis compound and its structure with his collaborators developing WZ-4-002, but did not share the physical material. The Judge, therefore found that Eck did not violate the plain terms of the MTA and under the MTA, Novartis could no claim priority over WZ-4-002.

Lessons Learned: Research funding from industry with expansive terms over potential research discoveries is problematic for research institutions. Narrower, project specific agreements are preferable, especially over discoveries that are far upstream from clinical application. Approach of the SGC in this context is preferable, placing few restrictions on protein structures and probes, enabling further development by a range of parties….

5. ***Orchid Biosciences v. St. Louis University, 2001***
- Orchid Biosciences Inc. v. St. Louis University, 198 F.R.D. 670 (S.D. Cal. 2001).

St. Louis University sought a declaration for non-infringement, invalidity and non-enforceability of a patent held by Orchid Biosciences over detection of genetic diseases and gene sequence variations by single nucleotide primer extension. The issue was whether there was a sufficient connection between the University and the State of California for courts in that State to have jurisdiction. MTAs, confidentiality agreements and clinical trial agreements that the University had with entities in California over two years were used as evidence of jurisdiction, and the court ordered their production during discovery.

[16] Dana–Farber Cancer Institute Inc. v. Gatekeeper Pharmaceuticals Inc. v. Dana–Farber Cancer Institute Inc. and Novartis Pharma A.G., Novartis International Pharmaceutical, Ltd., and Novartis Institutes for Biomedical Research, Inc., No. 10–11613, 2012 WL 4960172 (D. Mass. Oct. 12, 2012).

6. ***Clontech Laboratories, Inc. v. BCM Technologies, Inc., and Baylor College of Medicine, 2003***

- Baylor College of Medicine and BCM Technologies Inc. v. Clontech Laboratories Inc., No. H-02-0016 (S.D. Tex. 2002) [Trial decision, 2002]
- Baylor College of Medicine and BCM Technologies Inc. v. Clontech Laboratories Inc., No. H-02-0017 (S.D. Tex. 2003).

In 1998, Baylor and Clontech entered into a licensing agreement over cloning technology developed by Baylor-affiliated researcher, Dr. Stephen Elledge. Prior to these negotiations, Baylor had applied for a patent over the cloning technology. During the negotiations from July-December 1998, Baylor provided Clontech with trade secrets and confidential and proprietary information so that Clontech could better evaluate the technology. Information and materials were transferred under a Confidential Disclosure Agreement and an MTA, which limited Clontech's use to non-commercial evaluation and agreed not to use the information for prohibited commercial uses. Clontech also agreed that it would "promptly disclose to Baylor all inventions made through the use of the materials and assign any right to Baylor, subject to the right to a non-exclusive license to use the same for research purposes."[17] Baylor obtained a patent over the technology in Dec 1998 and informed Clontech that it was licensing the technology to another company. After the negotiations ended, Baylor alleged that Clontech used the contents of the pending patent and other proprietary information to develop and market a product called "Creator™" in violation of the two Agreements. Clontech insisted it used the materials provided solely for evaluating the scientific merit of the technology. It argued that the Creator system, which it began to commercially exploit about a year after the negotiations with Baylor failed, was a result of independent development efforts at Clontech and did not incorporate or include any information or materials received from Baylor. In November 2001, Baylor sued Clontech for breach of contract, misappropriation of trade secrets, conversion, breach of fiduciary relationship and statutory theft of trade secrets.[18] Clontech sought, among other things, a declaratory judgment that it had not breached Baylor's patent. The key decision was simply that the State Court had jurisdiction to issue such a declaration, but the case was procedural and not substantive. There are no further decisions and it appears that the case settled without a determination on the patents. The case was closed in April 2003.

[17] Baylor College of Medicine and BCM Technologies Inc. v. Clontech Laboratories Inc., No. H-02-0017 (S.D. Tex. 2003).

[18] Baylor College of Medicine and BCM Technologies Inc. v. Clontech Laboratories Inc., No. H-02-0017 (S.D. Tex. 2003)

Technology Transfer Entity versus Company

7. ***Yeda Research and Development v. Imclone Systems, 2006***

- Yeda Research and Development Co. Ltd. v. Imclone Systems Inc., and Aventis Pharmaceuticals Inc., 443 F.2d 570 (S.D.N.Y. 2006).

This dispute is about inventorship of a patent (US 6,217,866) that claims methods for combining a mono-clonal antibody (mAB) with chemo-therapy agents to synergistically target human tumor cells. The therapeutic effect of the combination was greater than either the mAB or chemotherapy alone. Research on the combination therapy had been conducted by three scientists at the Weizmann Institute of Science in Israel, the intellectual property of which is managed by Yeda Research and Development. The work on the combination therapy was based on a mAB, developed by Dr. Joseph Schlessinger, who in 1986, had accepted a sabbatical and then a position as Research Director at a US biotechnology company in Maryland, Meloy Laboratories Inc. Meloy Laboratories was sold to Rorer Biotechnology Inc., which merged to become Rhone Poulenc Rorer, and then merged again to become Aventis Pharmaceuticals, which is a wholly owned subsidiary of the Sanofi-Aventis Group. During the prosecution at the patent office of the patents in question, Rorer exclusively licensed the intellectual property and any resulting patents to ImClone Systems, Inc, which therefore controlled the patent that was eventually granted and was the subject of the litigation. This history and the patent prosecution, from filing to application, spanned 1988 to 2001. Listed as inventors on the patent were Schlessinger and other researchers who had worked with him at Meloy/Rorer. No scientists from the Weizmann Institute were listed as inventors.

Research at the Weizman Institute was led by immunologist Dr. Michael Sela and conducted by two researchers in his lab, Drs. Esther Aboud-Pirak and Esther Hurwitz. The goal was to develop conjugates between a carrier that could specifically target the growth of tumour cells via EGF receptors, so the team turned to Schlessinger to supply a mAB (108) that he and his team had developed at Meloy/Rorer. Sela and his team spent the next fourteen months working with the mAB, leading to the experiments that mixed mAb delivery with specific chemotherapy drugs. They drafted a manuscript on the work, which they shared and later co-authored with Schlessinger as the originator of the mAb. The companies, in drafting their patent, directly copied text and figures from that manuscript prior to its publication and did not include the Weizmann researchers as co-inventors. They overcame the paper as prior art by claiming that the Weizmann researchers conducted their experiments as contract researchers purely under the direction of the Schlessinger team, which had conceived of the invention. This version of facts was rejected by the Judge, who found no contemporaneous documentary evidence to support this assertion.

The patent, which issued after a protracted prosecution at the USPTO, contained only claims relevant to the method of using the mAb in combination, in general and for therapeutic purposes. In other words, the claims related solely to the work of the Weizmann researchers. Other claims

for the mAb itself were denied by the patent office as not novel, but ImClone attempted to achieve an earlier priority date for the invention to circumvent another mAb patent. This would necessitate a review of laboratory records, which were not forthcoming from Meloy or Rorer. Indeed, the judge stated that at least one of the listed inventors "did not keep organized notebooks, and in fact could not recognize much of the handwriting in her own folders, the Court was left without a clear picture of what experiments the named inventors performed, and in what order."[19] ImClone therefore turned to Sela to request notebooks from his lab, but instead received a reply from Yeda stating it knew nothing of a patent application. Thereafter, ImClone decided to "stonewall Yeda, claiming that the matter had become irrelevant in light of other developments."[20] But in 2002, Yeda discovered the patent and began to investigate the issue of inventorship, which lead to the current law suit.

After a three-week bench trial, the judge found that the invention narrative as related by Schlessinger and his team was not credible, and therefore found that the Weizmann researchers, and not the Schlessinger team, were the inventors of the patent. Jenie (2006) reported that following the decision ImClone was considering three options: (1) an appeal of the decision; (2) attempting to invalidate the patent; and (3) licensing the patent from Yeda. ImClone appealed the decision, but a settlement was reached and the case was dismissed in January 2008.[21] While settlement terms are confidential, the deal may be highly valuable to Yeda. When the patent issued, ImClone entered into an agreement with Bristol Myers Squibb (BMS) for the development, promotion, distribution and supply in the United States of Erbitux ®, an mAb similar to that used in the Weizmann experiments. Under this agreement, ImClone had obtained more than $1.3 billion from BMS in milestone and royalty payments.[22]

Some lessons may be taken from this case. The first is that Schlessinger transferred the mAb to researchers at the Weizmann and other laboratories with no MTA or collaborative research agreement in place. Schlessinger testified that he informed the Weizmann researchers that the mAb's were proprietary to Rorer, all commercial rights belonged to Rorer, Rorer would require review of any publications prior to submission and that the mAb's could not be transferred to third parties. However, the judge found that this and other parts of Schlessinger's testimony were not credible, especially since there was no signed MTA and it was unlikely that Sela would have

---

[19] Groombridge N, Gearing BP (2008) Practical Lessons from a "Made for TV" Patent Litigation: The Trial of *Yeda Research and Development Co. Ltd v. ImClone Systems Inc. and Aventis Pharmaceuticals Inc.* The Federal Lawyer Feb: 51-55.
[20] Groombridge N, Gearing BP (2008) Practical Lessons from a "Made for TV" Patent Litigation: The Trial of *Yeda Research and Development Co. Ltd v. ImClone Systems Inc. and Aventis Pharmaceuticals Inc.* The Federal Lawyer Feb: 51-55.
[21] Jenie S (2006) ImClone Loses patent due to lack of written records (and unclean hands). Available: http://www.patentbaristas.com/archives/2006/09/20/imclone-loses-patent-due-to-lack-of-written-records-and-unclean-hands/. Accessed 27 September 2014.
[22] Groombridge N, Gearing BP (2008) Practical Lessons from a "Made for TV" Patent Litigation: The Trial of *Yeda Research and Development Co. Ltd v. ImClone Systems Inc. and Aventis Pharmaceuticals Inc.* The Federal Lawyer Feb: 51-55.

performed research on the mAb under these terms. Indeed, there was no evidence of contract research. The fact that it was standard practice for Meloy/Rorer to require an MTA in the transfer of proprietary materials bolstered the conclusion of the Judge that "no such agreement existed and that Schlessinger did not place any restrictions on the Weizmann's use of the antibodies".[23] The second lesson is that researchers should always ensure that they are keeping good records of their research progress.

Disputes Between Universities and Their Employees

Cases 8-10

- Yeda Research and Development Co. Ltd. v. Imclone Systems Inc., and Aventis Pharmaceuticals Inc., 443 F.2d 570 (S.D.N.Y. 2006).
- Yeshiva University v. Greenberg, 681 N.Y.S.2d 71 (S Ct. Appellate Div. 2d Dept. N.Y. 1998).
- Yeshiva University v. Greenberg, 644 N.Y.S.2d 313 (S Ct. Appellate Div. 2d Dept. N.Y. 1996).
- Washington University v. Catalona, Not Reported in F.Supp.2d, 2006 WL 5190727 (E.D.Mo. 2006).
- Washington University v. Catalona, 490 F.3d 667 (8th Cir. 2007).

MTAs also feature in disputes between Universities and their research staff. In *United University Professions v. State of New York, State University of New York and Upstate Medical University*, the organization representing staff at the University fought to uphold the ruling of an arbitrator to reinstate an Associate Professor, Dr. Feuer, who was also the director of the Upstate Humanized Severe Combined Immunodeficiency ("SCID") Mouse Center and the Stem Cell Processing Center.[24] Feuer founded a company, HuMurine, that sold and shipped mice owned by Upstate Medical University, charged the shipping costs to a State grant contract, and failed to comply with the MTAs. Since he did not intend to profit personally, and believed his company would benefit the University and provide financial support for his research and the university administrators were aware of his actions, an arbitrator decided that a suspension, not dismissal was warranted.

In *Yeshiva University v. Greenberg*, a research assistant, Greenberg, was employed in the Pathology Department of Albert Einstein College of Medicine to work on the development of PHF-1, an antibody that can detect markers associated with Alzheimer's disease and a cell line that produces PHF-1. Greenberg left for new employment and took samples of both PHF-q and the cell line to her new position to continue her work. The College continued to use an MTA to distribute the antibodies and cell lines to third parties for non-commercial use. The College then learned that Greenberg was also distributing the lines, including to commercial entities. The Courts agreed with the College's interpretation of its employment contract, that it owned the

---

[23] Yeda Research and Development Co. Ltd. v. Imclone Systems Inc., and Aventis Pharmaceuticals Inc., 443 F.2d 570 (S.D.N.Y. 2006).
[24] United University Professions v. State of New York, 966 N.Y.S. 350 (2013).

antibody and cell line.[25] An interim decision granted an injunction to stop Greenberg from distributing the material because such distribution would be detrimental to its existing licensee, Molecular Geriatrics Inc, from developing commercial products, including diagnostic tests and therapeutics.[26]

In a well-known dispute between Washington University and respected urologist, William Catalona, over the ownership of biological samples donated by research participants for prostate cancer research to the Genito-urinary Biorepository, MTAs signed by Dr. Catalona were used as evidence that Washington University owned the biological samples.[27] Research participants therefore could not grant permission for Dr. Catalona to move their samples to his new position at Northwestern University. Their only remaining interest in the biological samples was the right to withdraw from research and prevent further use or ensure destruction of the samples according to the consent form that accompanied their deposit.

Corporations and Individual Researchers and their Research Institutions

11. *Rouse v. Walter & Assoc.*

- Rouse v. Walter & Assoc., 513 F. Supp.2d 1041 (S.D. Iowa 2007).

In *Rouse v. Walter and Assoc.*, 2007, Iowa State University researchers claimed copyright infringement for agricultural software for predicting beef quality against a consulting company, but did not possess valid copyright under their employment contracts.[28] The dispute was over licensing terms of the software and the terms of an MTA that transferred the rights in software to Iowa State University was part of the evidence that the researchers did not hold copyright in the software, even though the plaintiffs contended that the MTA “was just a boilerplate document use to transfer two programs to a joint research project”.[29]

12. *Davidson v Cao*

- Donald Davidson and Abbott Laboratories v. Yihai Cao, Judah Folkman, Michael S. O'Reilly, the Children's Medical Center Corporation and Entremed Inc., 211 F. Supp. 264 (D. Mass. 2002).

This suit arose out of a dispute between hospital and industry researchers over the inventorship of patents related to the Kringle 5 region of plasminogen to treat cancer and other angiogenic diseases, such as diabetic retinopathy. Abbott claimed that one of its researchers made the discovery of Kringle 5 and that the Boston Children’s Hospital unlawfully misappropriated and conspired to steal that invention. The Hospital researchers claimed that the suit was a false

---

[25] Yeshiva University v. Greenberg, 681 N.Y.S.2d 71 (S Ct. Appellate Div. 2d Dept. N.Y. 1998).
[26] Yeshiva University v. Greenberg, 644 N.Y.S.2d 313 (S Ct. Appellate Div. 2d Dept. N.Y. 1996).
[27] Washington University v. Catalona, 490 F.3d 667 (8th Cir. 2007).
[28] Rouse v. Walter & Assoc., 513 F. Supp.2d 1041 (S.D. Iowa 2007).
[29] Rouse v. Walter & Assoc., 513 F. Supp.2d 1041 (S.D. Iowa 2007).

attempt to intimidate the hospital and damage the reputation of its researchers.[30] The Hospital filed a “vicious” countersuit “for defamation and conspiracy, accusing the Illinois pharmaceutical giant of implementing a fraudulent scheme to obtain patent rights to anti-angiogenic agents studied in the laboratory of Judah Folkman.”[31]

Dr. Judah Folkman, at the Boston Children’s Hospital, was the first to propose that tumors require angiogenesis for growth. In early 1994, he and two fellows, Yihai Cao and Michael O’Reilly, in his laboratory began to study the anti-angiogenic properties of Kringle fragments in plasminogen. Cao focused his efforts on a novel Kringle 5, but needed additional plasminogen fragments. These were provided by Donald Davidson, a research biochemist at Abbott without a confidentiality agreement or an MTA. A year later, Davidson sent Folkman a confidential disclosure agreement (CDA) signed by Abbott stating that Abbott would provide the fragments subject to the full execution of the agreement. The broad terms that gave Abbott rights to any inventions made or developed as a result of “exposure to Abbott’s [fragments]”, and a six-month “exclusive option to purchase the Children’s Hospital share of any joint invention”. Folkman signed the CDA without reviewing it in detail and forwarded it the Hospital’s technology transfer office (TTO), which determined that the agreement was unacceptable. The TTO instead proposed an alternative based on the Uniform Biological Materials Transfer Agreement (UBMTA) that left the Hospital free to file patent applications and claim inventions based on the material supplied by Abbott. This resulted in a classic example of the “Battle of the Forms”, whereby each party sent variations of its own standard form agreements to the others, without the terms of any one form being agreed to by both parties. Agreement, or “meeting of the minds” on terms, is a prerequisite for a valid and enforceable contract.

Meanwhile, through summer and fall of 1995, Davidson continued to supply fragments, including the novel Kringle 5, to Cao without knowing that the Hospital had found the Abbott CDA unacceptable and Cao continued to share experimental results with Davidson.[32] By November, however, Davidson’s supervisor wrote to Folkman proposing a “gentlemen’s agreement” that Davidson would continue to supply Kringle fragments “with the understanding that the techniques and knowledge or [Folkman’s] lab would be readily available [to Abbott]” and that Davidson should visit Folkman’s lab.[33] Folkman declined this proposal, and, in December, Folkman and Cao filed a provisional patent on the use of Kringle 5 to inhibit cell growth (later granted as US 5,854,332). In January 1996, Abbott sent a different CDA signed by Abbott that prohibited the Hospital from asserting intellectual property rights and granted Abbott property rights in all information and developments based on Abbott Material. The Hospital TTO

---

[30] Brower V (2000) Fight for reputation. 301-302 p.
[31] Novak K (2000) Folkman countersuit attacks "fraudulent" Abbott. Nature Medicine 6: 948.
[32] Donald Davidson and Abbott Laboratories v. Yihai Cao, Judah Folkman, Michael S. O'Reilly, the Children's Medical Center Corporation and Entremed Inc., 211 F. Supp. 264 (D. Mass. 2002).
[33] Donald Davidson and Abbott Laboratories v. Yihai Cao, Judah Folkman, Michael S. O'Reilly, the Children's Medical Center Corporation and Entremed Inc., 211 F. Supp. 264 (D. Mass. 2002).

rejected this agreement, stating “there is some misunderstanding between the two parties” and asking Abbott to send an MTA under which the Hospital would promise not the share Abbott materials with third parties.[34] In May 1996, Abbott filed its own patents over Kringle 5, naming Davidson as inventor, however, the Hospital claimed that this patent was the direct result of information from Cao and that Davidson had told Cao he would be included as a co-inventor on any patent applications. These actions resulted in a dispute over the inventorship of the patents, but the disagreement went further. In 1997, Abbott insisted that the Hospital assign its patent to Abbott. The Hospital refused but entered into informal discussions with Abbott to resolve the inventorship dispute. In this attempt, the Hospital and Abbott exchanged confidential information, including lab notes, under a CDA signed in late 1997.

“During discussions, Abbott threatened to publicly embarrass Folkman if its demands were not met. The discussions ended in the early fall of 1998 without a resolution of the inventorship dispute.”[35] At this point, Abbott discussed the lawsuit with the press, including *The New York Times* and *The Boston Globe* as well as with scientific journals. In addition to a breach of contract suit based on the CDA and the inventorship dispute, this press coverage also resulted in a defamation suit brought by Folkman against Abbott. Commentary from *Nature* and *EMBO Reports* headlined “Angiogenesis team sued by pharmaceutical company” and “Fight for reputation” concluded that this suit would “certainly not be the end of lawsuits against scientists in an industry that is increasingly using courts to obtain control over important molecules. And given the publicity surrounding this case, scientists in academia may become less willing to cooperate with Abbott or other drug companies that may question their integrity in seeking profits from shared discoveries.” [36]

The case before the District Court was to dismiss some of the claims in the ongoing litigation, most of which were allowed to move forward.

Lessons Learned*:* In relations between public sector institutions and industry in the supply of materials, it is essential to clarify the terms of the transfer of materials prior to their research use. In this case, a negotiated MTA with reasonable terms that fairly recognize the contributions of the parties is essential and will likely prevent disputes down the road. Disputes damage research relationships and damage the reputations all actors involved.

[34] Donald Davidson and Abbott Laboratories v. Yihai Cao, Judah Folkman, Michael S. O'Reilly, the Children's Medical Center Corporation and Entremed Inc., 211 F. Supp. 264 (D. Mass. 2002).
[35] Brower V (2000) Fight for reputation. 301-302 p.
[36] Brower V (2000) Fight for reputation. 301-302 p.

13. ***E.I. Dupont de Nemours and Co v Okuley, 2000***

- E.I. Du Pont de Nemours and Co. v. Dr. John J. Okuley, No. C2-97-1205, 2000 WL 1911430 (S.D. Ohio Dec. 21, 2000)

This suit arose out of a Research Collaboration Agreement between Dr. John Browse, head of the Institute of Biological Chemistry at Washington State University ("WSU"), and Du Pont over inventorship of a series of patents and patent continuations. Du Pont sought to isolate and clone certain genes that would be useful in engineering crop plants yielding vegetable oil containing a higher quality or quantity of beneficial fatty acids and lower quantities of saturated fats. If successful, Du Pont hoped to patent these genes. The Research Collaboration Agreement, drafted by Du Pont, was in the form of a letter to Dr. John Browse, dated December 14, 1989. It was accepted and signed by Dr. Browse, and approved by WSU on December 15, 1989. Under the agreement, any Biological Materials remained the property of Du Pont, could only be used for research purposes in support of the collaboration, and third party transfer was prohibited. In addition, Du Pont had a first right to refuse an exclusive license for the commercial use of any secondary DNA isolated by Dr. Browse. Any ideas and improvements conceived during the term of the Agreement were to be communicated promptly to Du Pont and all rights were to be assigned to Du Pont, with some exceptions. DuPont did not fund the research, but provided access to a large library of biological material in the form of tagged Arabidopsis mutants. The agreement to assign ownership to Du Pont also covered individuals working in Dr. Browse's lab, including postdoctoral fellow, John Okuley. Okuley disputed this fact since he cloned the gene of interest while visiting a lab at Ohio State University. However, since he was still an employee of WSU, the agreement with DuPont covered his discovery of the gene and his assignment of rights to DuPont was valid. The court found it had no jurisdiction to decide on the inventorship issues because the patent had not yet issued, but decided that regardless, Okuley was obligated to assign all rights over his discoveries to DuPont based on both the patent policy of WSU and the agreement between Browse and DuPont to which WSU was a party. In addition, Ohio State University had waived any claims to inventions on this project made by Okuley while he was working in their facilities. Therefore, DuPont won on summary judgment that it held the ownership interest of the genes in question.

Lessons Learned: The Collaborative Research Agreement was not well drafted and not particularly clear on its coverage of individuals working under the supervision of the principal investigator. When staff, fellows, and students are working on research projects that are covered by agreements with industry partners, best practices are to clarify expectations around confidential information, use of materials, and resulting inventions. It is a good idea to have a "lab agreement" under which these terms and expectations are acknowledged by all members of the lab.

Individuals against Corporations and Research Institutions

Cases 14-16.

- Meco v. Novaspes Inc., 2006 WL 93245 (Cal. App. 2 Dist. 2006).
- American Type Culture Collection (ATCC) v. Coleman, 26 S.W.3d 37 (C.A. Tex 1st Dist 2000).
- Holzina v. Boston Scientific Corp., 2013 WL 1411656 (S.D.W.Va.).

Meco v. Novaspes Inc, 2006, arose from the US sales distribution of herbal dietary supplements (PC-Spes) that were allegedly illegally advertised to cure prostate cancer and other serious illnesses. According to plaintiffs in this class action lawsuit, the supplements were secretly adulterated with prescription drugs and caused serious side effects and deaths. California health authorities ultimately recalled them from the market. However, this litigation was about whether Californian courts had jurisdiction over the dispute. Caught in the mesh of litigation was the New York Medical College because the developer of the supplements, Sophie Chen, was a faculty member. The College, through a Collaboration Research Agreement (governed by New York law), contracted with International Medical Research (IMR), a Californian company, to investigate the herbal action against prostate cancer of PC-Spes and its compounds. The compounds were transferred to the College under an MTA, which also prohibited the College or its investigators from providing the materials to other parties without IMR's consent. The agreement did not prohibit publication, but required 45 days for IMR review. The research resulted in publications in peer-reviewed journals, including the *New England Journal of Medicine*, but these could not be construed as marketing aimed at Californians. There was also no evidence that the College intentionally falsified or misconducted its research, especially on side-effects, in a manner that conferred a benefit on IMR or caused a detrimental effect in California. None of the links between IMR and the College were sufficient to confer jurisdiction of California courts over the College.

In *American Type Culture Collection (ATCC) v. Coleman*, a group of Gulf War veterans and their families brought an action against ATCC, a non-profit repository and distribution center for microbes, viruses and cell lines, alleging that it had sold dangerous pathogens to Iraq that were then used in weapons against veterans during the Gulf War.[37] The issue in the 2000 hearing was whether Texas courts had jurisdiction over the suit, meaning that there had to be a connection to Texas for this national mailorder company incorporated in the District of Columbia and operating in Maryland. Material Transfer Agreements with Texas institutions were weak evidence that ATCC had a business presence in Texas, but along with other evidence of contracts and activities in Texas, the court ruled that ATCC had continuous and systematic contacts with Texas to grant personal jurisdiction over ATCC. In *Holzina v. Boston Scientific*, the court

[37] American Type Culture Collection (ATCC) v. Coleman, 26 S.W.3d 37 (C.A. Tex 1st Dist 2000).

reached the opposite conclusion, an MTA between two companies was not sufficient to establish that the defendant had a connection with Georgia in this personal injury suit.[38]

Criminal—Mail Fraud

17. *U.S. v. Kurtz*

- United States of America v. Steven Kurtz, No. 04-CR-0155A, 2008 WL 1820903 (W.D.N.Y Apr. 21, 2008).

The ATCC was involved in further litigation, and the only criminal case that we found in our search. This case sought to dismiss an indictment for mail fraud against a faculty member. Steven Kurtz, of the State University of New York at Buffalo, who obtained biological materials via a collaborator, Robert Ferral, at the University of Pittsburgh (UP) Human Genetics Laboratory. ATCC only supplied biological materials to approved and registered business and institutions, including UP. Ferall ordered the biological materials using UP's account and then supplied them to Kurtz, in violation of an MTA that prohibited transfer of materials to third parties. The Magistrate dismissed the indictment because ATCC was paid for the biological materials, albeit by UP, and there was no evidence that the scheme depended on a misrepresentation of an essential element of the MTA. The restriction on third party transfer was not an essential element of the bargain, and the indictment did not allege that either Kurtz or Ferrell "even know about the transfer restriction".[39]

Corporation versus Corporation

18. *W.L. Gore & Assoc v GI Dynamics Inc 2010 and 2012*

- W.L. Gore & Assoc. v. GI Dynamics, Inc., 2010 WL 5184254 (D. Ariz 2010).
- W.L. Gore & Assoc. v. GI Dynamics, Inc., 872 F. Supp.2d 883 (D. Ariz 2012).

This dispute was over efforts to develop an intestinal sleeve device anchored in the gastro-intestinal tract to assist patients suffering from morbid obesity and/or Type II diabetes. The companies entered into a series of CDAs and an MTA to cover samples of small porosity, high bubble point ePTFE tubes to GID for evaluation through *in vitro* and animal studies. The MTA also contemplated a long-term supply agreement, pending successful completion of certain milestones. Later, Gore entered into a Supply Agreement with GID for the tubes and GID agreed to make milestone payments and royalties from sales of products manufactured from the tubes. The dispute arose over differing interpretations of the CDAs and the MTA as to which party had the rights to make intestinal sleeve devices. Gore understood the MTA to grant it rights to make, use and sell any material disclosed under the MTA to any party in any form for any use,

---

[38] Holzina v. Boston Scientific Corp., 2013 WL 1411656 (S.D.W.Va.).
[39] United States of America v. Steven Kurtz, No. 04-CR-0155A, 2008 WL 1820903 (W.D.N.Y Apr. 21, 2008).

including sleeves as incorporated in completed intestinal sleeve devices. GID, on the other hand, asserts that Gore does not have a right to make intestinal sleeve devices covered by GID's patents.

19. *DSM Research v. Verenium Corp, 2010*

- DSM Research v. Verenium Corp., 686 F.Supp.2d 159 (D. Mass. 2010).

A company based in the Netherlands filed suit on 12 January 2009 against a Massachusetts-based company with operations in California for unauthorized use of confidential information and trade secrets. The civil action was to transfer the case from Massachusetts to California, but that was denied due to inconvenience to the European corporation. The two companies had entered into a one-year MTA through which DSM transferred a microbial strain for R&D into biofuels to Diversa, which later merged with a second company to become Verenium Corporation. The two companies attempted negotiations for licensing deals but the agreements were never finalized. DSM alleged that Verenium proceeded to develop the microbial strain commercially. The case closed on 8 April 2010, but it is unclear if settlement was reached.

20. *Cancer Genetics Inc v Kreatech Biotechnology*, 2007

- Cancer Genetics Inc. c. Kreatech Biotechnology, 2007 WL 4365328 (D.N.J.).

This case was also to transfer from New Jersey to the Southern District of New York. Both companies, Kreatech based in Amsterdam and Cancer Genetics based in New Jersey were biotechnology companies that developed genomic-based products to diagnose and monitor cancer. CGI produced probes and Kreatech labelled the probes for CGI under a License Agreement that expired in 2004. In 2006, CGI, Kreatech and a Spanish company Master Diagnostica, entered into a Letter of Understanding and an MTA, which enabled Kreatech to label CGI probes and for Master Diagnostica to distribute the labeled probes in Europe. Both the LU and the MTA contained a jurisdiction clause for New York. Probe sales were less than anticipated and CGI owned Kreatech approximately $300,000. CGI failed to make a scheduled payment and instead filed a complaint that Kreatech retained CGI products and, contrary to the various agreements, including the MTA, which required return of the products, continued to use, manufacture and market probes in competition with CGI. CGIs action was for breach of contract and fraud arising out of the LU and MTA and Kreatech's counterclaim was over CGI's defaulted payments. The case was transferred to the New York Southern district, settled, and was closed on 26 June 2007, without a disposition (PACER).

21. *Unigen Pharmaceuticals v Colgate Palmolive, 2007*

- Unigen Pharmaceuticals v. Colgate-Palmolive Co., 2007 WL 2671025 (W.D. Wash. 2007)

A claim for breach of contract under Colorado law, which alleges that "Colgate, in breach of the NDA and MTA, misappropriated, used, and disclosed Unigen's Confidential Information without the consent of Unigen." Unigen developed, manufactured and sold therapeutic compounds, including "Univestin". Unigen claimed it invented a new use for Univestin, namely to use the product in an oral formulation to promote gum and oral health. It therefore contacted Colgate Palmolive, a leading manufacturer of oral care products, to discuss a possible collaboration and commercialization opportunities. The parties entered into a non-disclosure agreement under which Colgate could use Unigen's proprietary information for limited purposes and prohibited the parties from publishing or disclosing to any third parties any of the confidential information without the prior consent of the other party. On May 24, 2004, the parties also entered into an MTA, which provided that Unigen's biological or chemical materials to be transferred to Colgate were to be considered confidential and proprietary, were to be used only for noncommercial research and evaluation purposes, and were to be retained in confidence and not disclosed without Unigen's prior written consent. Both the NDA and MTA contained Colorado choice of law provisions. Unigen alleged a breach of both the NDA and the MTA because Colgate filed a provisional patent application, naming only Colgate employees as inventors, directed towards the use of Univestin in an oral care composition, relying on Unigen's confidential information. The one published decision was about the jurisdiction of the court over contractual matters relating to a patent application. There are no further records of the dispute.

22. *Bavarian Nordic v. Acambis Inc, 2007*

- Bavarian Nordic v. Acambis Inc., 486 F.Supp.2d 354 (D. Del. 2007).

This case is between a two companies, with the plaintiff company, Bavarian Nordic joined by a German research professor, Dr. Anton Mayr. The plaintiff's claimed tortious interference and unfair competition over the development of vaccinia-virus based vaccines. Mayr's research dated back to 1955 when he worked at the Bavarian State Vaccination Institute under the supervision of Professor Herrlich on the development of a safer smallpox vaccine from a vaccine virus strain deposited at the Ankara Vaccine Institute (CVA virus). Mayr passaged the CVA virus in non-host chicken fibroblast cells over 500 times to create an attenuated virus. The Free State of Bavaria filed a German patent application over it in 1971.

By 1974, Mayr was the director of the Institute for Medical Microbiology, Infectious and Epidemic Diseases, Veterinary Faculty, University of Munich and co-authored two papers on the virus, now designated as the Modified Vaccinia Virus Ankara (MVA). MVA was tested in animals and for clinical use, but no documents existed on them or where the particular strain – MVA 572 – was created and there was no declaration that Mayr "owned" the virus. By the mid-

1990’s, however, Mayr was internationally recognized as the go-to source for MVA, and he shared it widely.

At that time, working in Mayr’s laboratory as a graduate student was Dr. Gerd Sutter, who developed MVA-F6 by further passaging of MVA 572. When Sutter moved to work with Dr. Bernard Moss at the National Institutes for Health (NIH), he brought MVA-F6 isolate with him. After further research, Sutter and Moss reported that MVA-F6 expressed both vaccine viral and recombinant proteins at a high level in non-permissive human cells and suggested that MVA would make a safe and efficient vector for vaccines, but the F6 isolate was laboratory grade. In 1995, Moss contacted Mayr for original isolates of MVA for the purpose of producing recombinant vaccines for clinical use. Mayr sent the samples, accompanied by a letter that placed no restrictions on use or transfer. By 1996, Sutter began working on the F6 strain with Bavarian Nordic, and Mayr entered into a consulting agreement with that company. That agreement gave Bavarian Nordic “the exclusive and sole access to MVA vaccine stock and MVA viral stock in the possession of Mayr”, but in recognition of non-commercial research interests, Bavarian Nordic agreed “not to unreasonably use its exclusivity to the MVA system to hinder basic research by third party non-commercial academia including the MVA system by rejecting access to the MVA system”.[40] However, the agreement also stated that Bavarian Nordic and Mayr could make no public announcement about the agreement. As stated by the judge, “Bavarian Nordic did not conduct any extensive due diligence in order to confirm that Dr. Mayr “owned” the “MVA system”, and according to Mayr, he did not read the agreement before signing it, having been assured that it was in his favour.[41]

In 2001, further correspondence between Mayr and Moss placed no restrictions on the use of the viral material shipped from Germany to the NIH. Then in July 2002, the NIH wrote a letter to Bavarian Nordic summarizing its understanding of the agreement between Mayr and Moss. The letter confirmed NIH intentions to use and make available to other parties derivative materials that had been developed by Moss. In August, NIH issued a Request for Proposals (RFP) for the “Development and Testing of a Modified Vaccinia Ankara (MVA) Vaccine” under which NIH provided access to “a master seed stock of MVA via an MTA. The NIH awarded the first two RFPs to Bavarian Nordic and Acambis Inc., and Bavarian Nordic brought this suit against Acambis Inc., but not the NIH. By letter in Nov 2002, Mayr wrote to Moss expressing his concern over the commercialization of MVA by the NIH, since his view was that he had distributed the strains to others for “strictly non-commercial purposes”.[42]

The action was for summary judgment in conversion – that Acambis Inc had “wrongfully exercised ownership of, or control or dominion over, personal property to which he has no right

[40] Bavarian Nordic v. Acambis Inc., 486 F.Supp.2d 354 (D. Del. 2007).
[41] Bavarian Nordic v. Acambis Inc., 486 F.Supp.2d 354 (D. Del. 2007).
[42] Bavarian Nordic v. Acambis Inc., 486 F.Supp.2d 354 (D. Del. 2007).

of possession at the time".[43] However, the Judge found that the facts "did not fit comfortably into the standard paradigm of a conversion claim". Neither Bavarian Nordic nor Mayr ever had physical possession of the specific material provided by the NIH to Acambis Inc. In addition, they had no right to immediate possession in the converted asset because there was no evidence that Mayr "owned" MVA due to his extensive collaborations and the ownership interests of the Government Agency for which he worked for part of his career. Finally, Mayr transferred the MVA 572 to the NIH without any restrictions on use or third party transfer, any patent rights had expired, and Mayr had widely distributed the material to the international research community for years. Claims for Unfair Competition under both State and Federal law failed equally and the court granted summary judgment in favour of the defendants.

Lessons: It is too late to try and the shut the gate once the horse has bolted. Property rights are weak when no patents have been issued.

23. *Alphamed Pharmaceuticals v Arriva Pharmaceuticals, 2005*

- Alphamed Pharmaceuticals Corp. v. Arriva Pharmaceuticals Inc., 2005 WL 5960935 (S.D.Fla 2005).

This case was over unfair competitive practices, interference with investors and breach of trade secrets. "AlphaMed is a biotechnology engineering and manufacturing firm, involved in the development and production of Alpha 1-Antitrypsin ("AAT"), a therapeutic protein produced naturally by the liver, which is released into the blood stream to protect tissue cells from damage caused by enzymes produced as the result of infection or inflammation within the. AAT is extremely useful for treating a wide range of human and veterinary indications, and pharmaceutical companies all over the world have undertaken significant research efforts to develop a method for the mass production of bio-synthetic AAT. Arriva is also a biopharmaceutical company, and its main corporate mission at the time of its creation was to develop a genetically engineered version of the drug Prolastin for use in pulmonary and topical applications."[44] AlphaMed claimed that Arriva has engaged in corporate espionage against AlphaMed in an attempt to develop the intellectual property necessary for exploitation of the market for AAT, including obtaining AlphaMed's trade secrets. One of the allegations was that Arriva pulled confidential documents, including MTAs from AlphaMed's trash. In addition, Arriva attempted to have AlphaMed investigated by the FBI, which resulted in AlphaMed's main investor pulling out of further support. The court did not grant summary judgment on any of the issues and remanded the case to a jury trial.

[43] Bavarian Nordic v. Acambis Inc., 486 F.Supp.2d 354 (D. Del. 2007).
[44] Alphamed Pharmaceuticals Corp. v. Arriva Pharmaceuticals Inc., 2005 WL 5960935 (S.D.Fla 2005).

NGO versus Government Department

24. *Edmonds Institute v. US Department of Interior, 2005*

- Edmonds Institute v. United States Department of the Interior, 383 F.Supp.2d 105 (D.C. 2005).
- Edmonds Institute v. United States Department of the Interior, Civil Action No. 04-1560 Memorandum Opinion (D.C. 2006).

A conservation organization brought an action under the Freedom of Information Act against the United States Department of the Interior seeking documents from the National Park Service relating to benefit sharing agreements with private parties that permitted the collection and commercial use of biological materials from national parks.
